# Oncological Outcomes of Patients With Different Pathological Features of pT3a Renal Tumor: A Systematic Review and Quantitative Synthesis

**DOI:** 10.3389/fonc.2021.678459

**Published:** 2021-06-03

**Authors:** Pengju Guo, Yongxing Wang, Yili Han, Dechao Wei, Jiahui Zhao, Mingchuan Li, Yongguang Jiang, Yong Luo

**Affiliations:** Department of Urology, Beijing Anzhen Hospital, Capital Medical University, Beijing, China

**Keywords:** renal tumor, pT3a, nephrectomy, cancer-specific survival, systematic review

## Abstract

**Purpose:**

To identify the differences in oncological outcomes for patients with different pT3a renal tumor invasion patterns and pathological features.

**Methods:**

The protocol of this study was registered on PROSPERO (CRD42021234475). Relevant studies were identified by searching the PubMed, Cochrane library, Embase, and Web of Science databases. Cancer-specific survival (CSS) was selected as the endpoint. Pooled hazard ratio (HR) and 95% confidence interval (CI) extracted from multivariate Cox models were evaluated to identify the hazard association.

**Results:**

A total of 22 studies, which enrolled 12384 patients were included for quantitative synthesis. Sinus fat invasion (SFI) + perinephric fat invasion (PFI) was associated with inferior CSS compared to SFI only (p = 0.02). Comparable CSS was observed between SFI and PFI (p = 0.57). SFI ± PFI showed inferior CSS compared to PFI only (p = 0.0002). The presence of pelvicalyceal system invasion significantly increased the risk of cancer-specific mortality (p = 0.0005). Renal vein invasion (RVI) indicated poor oncological outcomes in terms of CSS (p = 0.002). The concomitant RVI and fat invasion (FI) significantly increased the risk of deterioration of CSS compared to RVI or FI (p < 0.0001). Multiple invasion patterns translated into a significantly decreased CSS (p < 0.0001). Aggressive tumor behavior, including lymph node involvement (p = 0.006), distant metastases (p < 0.00001), sarcomatoid differentiation (p < 0.0001), necrosis (p < 0.0001), Fuhrman grade III or IV (p < 0.0001), positive margin (p < 0.0001), and tumor size >7cm (p < 0.0001) were the predictors of inferior CSS. The lymphovascular invasion (p = 0.67) was indolent in terms of CSS.

**Conclusion:**

This study confirmed the heterogenicity of pT3a renal tumors. Multiple invasion patterns could translate into a significantly decreased CSS, and SFI should not be merged in the SFI + PFI group. The presence of PSI or RVI could significantly increase the risk of cancer-specific mortality. Lymph node involvement, distant metastases, sarcomatoid differentiation, necrosis, high Fuhrman grade, positive margin, and size >7cm were the predictors of inferior CSS. A precise-risk grade of CSS for different invasion patterns including comprehensive combinations may be useful for the further refinements of the TNM system.

**Systematic Review Registration:**

The current study was registered on PROSPERO, and the registration numbers is CRD42021234475.

## Introduction

Since the publication of the sixth edition of the TNM staging system for renal tumors, the classification of T3a renal tumors has undergone several modifications. Although currently pT3a is defined as a tumor confined to the Gerota’s fascia but exhibiting perinephric fat invasion (PFI), sinus fat invasion (SFI), renal vein invasion (RVI), or/and pelvicalyceal system invasion (PSI) regardless of tumor diameter, a realistic controversy is whether pT3a represents a heterogeneous histological group where different elements or a combination may indicate a significant difference in oncological prognosis. The EAU guidelines on renal cell cancer (RCC) state that tumors with SFI might be more aggressive than tumors with PFI, which was consistent with the findings of Thompson et al. (1, 2). However, several studies evaluating oncological outcomes for different pT3a renal tumor invasion patterns have failed to demonstrate the significant difference (3, 4). Lack of consensus on the outcomes of different extrarenal extension patterns may result from the unstandardized definitions for the histological assessment of fat invasion in the early years and discrepancies in study design (2, 5).

Our understanding of the heterogeneous behavior of renal tumors has been well advanced. The increasing interest in adjuvant treatment, immunotherapy and targeted therapies has prompted the need for more accurate staging of renal tumors (5–9). In the clinical context, some pT3a renal tumors are confirmed by postoperative pathology, and their incidence is usually underestimated. Therefore, it is important to accurately predict the prognosis of different pT3a renal tumor invasion patterns to guide the follow-up protocols and evaluate the effect of postoperative therapies on survival. Given the continuing controversy over pT3a renal tumor staging, we undertook a systematic review and quantitative synthesis to determine whether pT3a represents a heterogeneous histological group and evaluate the oncological outcomes for different pT3a renal tumor invasion patterns and pathological features.

## Methods

This study was conducted according to the Preferred Reporting Items for Systematic Reviews and Meta-analysis (PRISMA) criteria, and the protocol was registered on PROSPERO for the study (CRD42021234475).

### Search Strategy

We searched the PubMed, Cochrane Library, Embase, and Web of Science databases for studies investigating the oncological outcomes of pT3a renal tumors from database inception to April 2021, using search terms integrated subject relevant terms (“renal tumor”, “renal neoplasm,” “renal cancer,” and “renal cell carcinoma”) and staging terms (“T3a,” “pT3a,” “T3,” “pT3,” “renal vein invasion,” and “urinary collecting system”). We also reviewed the references cited in the relevant articles to avoid omissions. The detailed Population, Intervention, Comparison, Outcome and Study design (PICOS) framework of the review was shown in **Table 1**. Only articles written in the English language were searched. All retrieved references were independently screened by two investigators (PG and YW) independently. When discordant decisions occurred, the senior authors (YJ and YL) were consulted to make final decisions.

**Table 1:** The Population, Intervention, Comparison, Outcome and Study design (PICOS) framework of the review.

Parameter	Inclusion criteria
Population (P)	Patients with pathological T3a and clinical or pathological N0-1M0-1 renal tumor.
Intervention (I)	Partial or radical nephrectomy
Comparison (C)	The cancer-specific survival of patients with different tumor invasion patterns or pathological features.
Outcome (O)	Cancer-specific survival with the hazard ratio (HR) and corresponding 95% confidence interval (CI) or p values in the multivariate cox models.
Study design (S)	Randomized trials, population-based, single and multi-center observational studies, and retrospective studies which were published in English.

### Study Selection, Data Extraction, and Quality Assessment

The studies on the oncological outcomes for different pT3a renal tumor invasion patterns and pathological features following partial or radical nephrectomy (PN or RN) were included. Cancer-specific survival (CSS) was considered as the single endpoint of oncological outcomes. Conference abstracts, reviews, commentary, editorials, and letters were excluded but checked for cited references. The studies that did not provide CSS with a hazard ratio (HR) and corresponding 95% confidence interval (CI) or p value in the multivariate cox models were also excluded.

Two investigators independently extracted the data from each study. Extracted data included the name of the first author, year of publication, recruitment period, country or region, study type, sample size, surgery types, and size of pathological features of different pT3a renal tumor invasion patterns. HRs and 95% CIs for CSS associated with different pT3a renal tumor invasion patterns and pathological features were extracted for quantitative synthesis. The quality of included studies was assessed using the Quality In Prognosis Studies (QUIPS) tool (10). The six bias domains when evaluating the literatures were study participation, study attrition, prognostic factor measurement, outcome measurement, study confounding, and statistical analysis and reporting. According to the items and considerations, the overall rating assessments were divided into low, moderate, and high risk of bias for each bias domain.

### Quantitative Synthesis and Analysis

The comparisons of CSS between different tumor invasion patterns were evaluated by the pooled HRs with corresponding 95% CIs. The statistical heterogeneity among studies was evaluated by the Cochrane Q test and quantified by I^2^ value. I^2^ ≤ 50% indicated no or moderate heterogeneity, and a fixed-effect model was applied. On the contrary, I^2^ > 50% indicated obvious heterogeneity, and a random-effect model was applied. The quantitative synthesis of HRs was evaluated by the inverse variance technique, and the quantitative synthesis of risk differences was evaluated by the Mantel-Haenszel test. The sensitivity analysis was conducted by one-removed analysis. Based on the recommendations of the Cochran manual, the evaluation of publication bias was conducted by using Egger’s test only when there were 10 or more included studies (11). The certainty of the evidence were evaluated according to the Grading of Recommendations Assessment, Development, and Evaluation (GRADE) approach, which yields four levels of evidence (i.e., very low, low, moderate, and high) (12). All statistical tests were performed using Review Manager 5.4 (Cochrane Collaboration, Oxford, UK) and Stata 15.1 (StataCorp., College Station, Texas). Statistical significance was set at p < 0.05, and all specified p values were two-sided.

## Results

**Figure 1:**
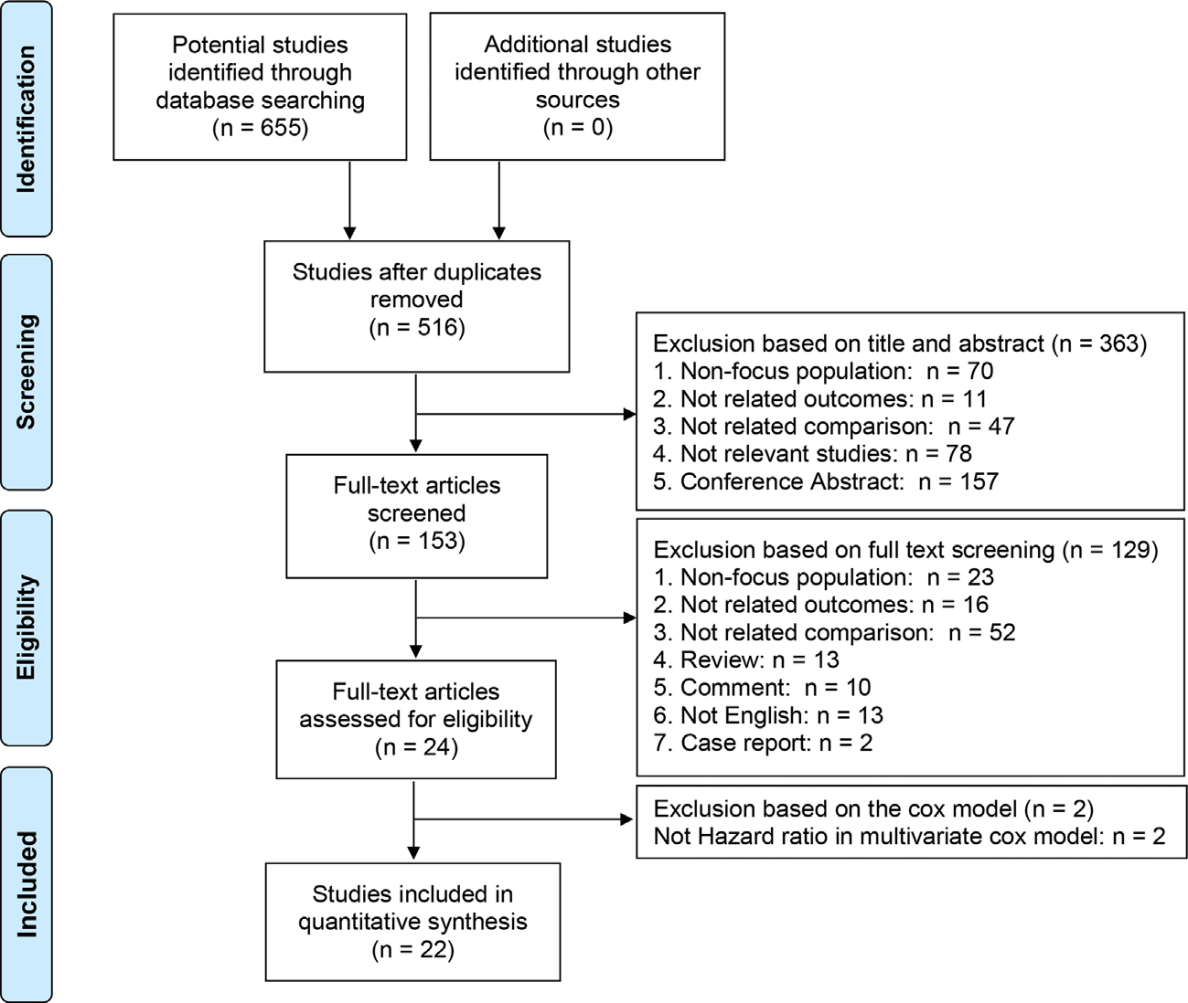
Flow diagram of study selection.

Among the 655 potential studies that were identified from the aforementioned databases, 139 studies were excluded due to duplication. After screening the titles and abstracts, 363 studies were found to be unsuitable for full text screening and were excluded. Based on the inclusion criteria, we conducted the full-text evaluation of the remaining 153 studies, among which 23 studies did not focus on the patients with pT3a renal tumor, 16 studies did not report the HRs of the CSS, 52 studies did not compare the CSS for patients with different invasion patterns or pathological features, 13 studies were reviews, 10 study were comments, 13 studies were not published in English, and two studies were case reports. Among the 24 full-text articles assessed for eligibility, two studies that only reported the relevant HRs in the univariate Cox models were also excluded. Finally, 22 studies enrolling 12,384 patients were included in the quantitative synthesis (**Figure 1**) (2–7, 13–28)

**Figure 2:**
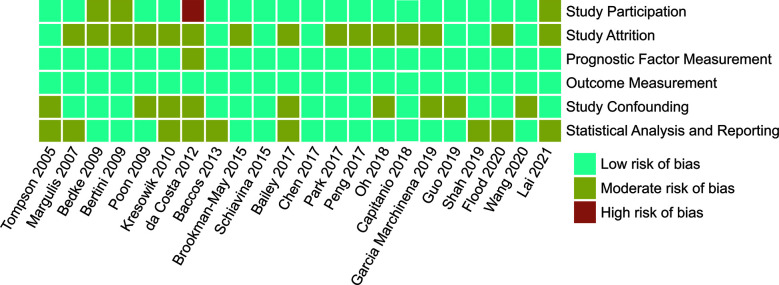
Risk of Bias for each enrolled studies assessed by QUIPS (Quality In Prognosis Studies) tool.

The enrolled studies were published between 2005 and 2021, and included 12 (54.5%) studies published over the past five years. All the enrolled studies were retrospective, and the recruited patients had no direct ipsilateral adrenal invasion. The recruited patients were from Asia, Europe, and USA. Ten studies focused only on patients following RN, 12 studies included patients who received PN or RN (**Table 2**). Using the QUIPS tool, the risk of bias for each enrolled study was assessed and the results are shown in **Figure 2**. Also, two enrolled studies used data from the Surveillance, Epidemiology, and End Results (SEER) database (6, 18). However, due to the recruitment period and the fact that most reported pathological features were different, it was reasonable to include these two studies. When two studies reported the endpoints of the same pathological features, we selected the most recent outcomes, i.e., those reported by Wang et al., because of the enrolled patients identified between 2010 to 2016 (6).

**Table 2:** Characteristics of the included studies.

Author	Year	Country	Recruitment period	Study design	Institution	Stage	Surgery type	Outcome	Cox model	Follow-up (mo)
Thompson (2)	2005	USA	1970–2002	RTP	Single	N0-1 M0-1	RN	CSS	Multi	72(24–408)*
Margulis (13)	2007	USA	1990-2006	RTP	Single	N0-1 M0-1	PN, RN	CSS	Uni, Multi	33.5(6.1–158.6)*
Poon (4)	2009	USA	1988–2007	RTP	Multiple	N0-1 M0-1	PN, RN	CSS	Uni, Multi	24(9–48)*
Bedke (14)	2009	Germany	1990–2007	RTP	Single	N0-1 M0-1	RN	CSS	Uni, Multi	34.8(14.4–109.2)*
Bertini (15)	2009	Italy	1989–2006	RTP	Single	N0-1 M0-1	RN	CSS	Uni, Multi	38(2–240)*
Kresowik (16)	2010	USA	1997–2007	RTP	Single	N0-1 M0-1	PN, RN	CSS	Multi	25.3(0–96.4)**
Chen (24)	2017	China	2006–2015	RTP	Single	N0 M0	RN	CSS	Uni, Multi	31 (3.4–109.7)
Park (17)	2017	South Korea	1997–2012	RTP	Single	N0 M0	PN, RN	CSS	Multi	58.1(37.2–86.5)*
Guo (18)	2019	China	1979–2014	RTP	Multiple	N0 M0	RN	CSS	Uni, Multi	NA
Shah (5)	2019	USA	1970–2011	RTP	Single	N0 M0	RN	CSS	Uni, Multi	111.6(81.6–160.8)*
Wang (6)	2020	China	2010–2016	RTP	Multiple	N0-1 M0-1	PN, RN	CSS	Multi	24(10–46)*
da Costa (19)	2012	Brazil	1992–2009	RTP	Single	N0-1 M0-1	PN, RN	CSS	Multi	28.6(3–60)**
Baccos (20)	2013	Italy	2000–2011	RTP	Single	N0-1 M0-1	RN	CSS	Uni, Multi	31(12–68.2)*
Flood (21)	2020	Canada	2011–2017	RTP	Single	N0-1 M0-1	RN	CSS	Multi	33.8(20.6–55.4)*
Schiavina (22)	2015	Italy	2000–2013	RTP	Single	N0-1 M0	RN	CSS	Uni, Multi	32(18–62)*
Brookman-May (3)	2015	Germany	1992–2010	RTP	Multiple	N0 M0	PN, RN	CSS	Multi	NA
Peng (23)	2017	China	2007–2012	RTP	Single	N0 M0	PN, RN	CSS	Multi	35.5(10–86)*
Oh (7)	2018	South Korea	1988–2015	RTP	Multiple	N0 M0	PN, RN	CSS	Uni, Multi	38.8(NA)**
Capitanio (25)	2018	Italy	1988–2015	RTP	Multiple	N0 M0	PN, RN	CSS	Multi	52(NA)**
Bailey (26)	2017	USA	2001–2010	RTP	Single	N0-1 M0-1	RN	CSS	Uni, Multi	98.4 (72–129.6)*
Garcia Marchinena (27)	2019	Argentina	2000–2016	RTP	Multiple	N0 M0	PN, RN	CSS	Uni, Multi	21(1–194)*
Lai (28)	2021	China	2000–2018	RTP	Single	N0-x M0	PN, RN	CSS	Uni, Multi	48(NA)*

Author	Year	No.pT3a	No.PFI only (%)	No.SFI only (%)	No.SFI+PFI (%)	No.SFI ± PFI (%)	No.FI (%)	No.RVI ± FI (%)	No.RVI+FI (%)	No.RVI only (%)	No.PSI (%)
Thompson (2)	2005	205	162 (79.0)	16 (7.8)	27 (13.2)	43 (21)	NA	NA	NA	NA	NA
Margulis (13)	2007	365	199 (54.5)	96 (26.3)	70 (19.2)	166 (45.5)	NA	NA	NA	NA	331 (90.7)
Poon (4)	2009	230	167 (72.6)	NA	NA	63 (27.4)	NA	NA	NA	NA	NA
Bedke (14)	2009	106	58 (54.7)	27 (25.5)	21 (19.8)	48 (45.3)	NA	NA	NA	NA	NA
Bertini (15)	2009	105	70 (66.7)	16 (15.2)	19 (18.1)	35 (33.3)	NA	NA	NA	NA	NA
Kresowik (16)	2010	110	36 (32.7)	41 (37.2)	33 (30)	74 (67.3)	NA	NA	57 (51.2)	NA	NA
Chen (24)	2017	163	79 (48.5)	11 (6.7)	NA	NA	NA	87 (53.4)	NA	NA	40 (24.5)
Park (17)	2017	266	92 (34.6)	51 (19.2)	29 (10.9)	80 (30.1)	172 (64.7)	94 (35.3)	69 (25.9)	25 (9.4)	NA
Guo (18)	2019	1869	687 (36.8)	381 (20.4)	105 (5.6)	486 (26.0)	1173 (62.8)	NA	696 (37.2)	NA	NA
Shah (5)	2019	563	114 (20.2)	51 (9.1)	NA	NA	NA	NA	NA	163 (29.0)	NA
Wang (6)	2020	5290	2569 (48.5)	1975 (37.3)	746 (14.1)	NA	NA	NA	NA	NA	NA
da Costa (19)	2012	46	NA	NA	NA	NA	24 (52.1)	NA	11 (23.9)	11 (23.9)	NA
Baccos (20)	2013	122	NA	NA	NA	NA	63 (51.6)	59 (48.4)	41 (33.6)	18 (14.8)	NA
Flood (21)	2020	160	NA	NA	NA	NA	NA	97 (61)	NA	NA	24 (15)
Schiavina (22)	2015	185	NA	NA	NA	NA	NA	NA	NA	NA	NA
Brookman-May (3)	2015	1247	NA	NA	NA	NA	1036 (83.1)	211 (16.9)	NA	NA	NA
Peng (23)	2017	125	NA	NA	NA	NA	89 (71.2)	NA	NA	36 (28.8)	NA
Oh (7)	2018	211	NA	NA	NA	NA	124 (58.8)	87 (41.2)	47 (22.3)	40 (19.0)	NA
Capitanio (25)	2018	309	164	68	NA	NA	NA	NA	NA	NA	NA
Bailey (26)	2017	325	NA	NA	NA	NA	NA	NA	NA	NA	27 (8.3)
Garcia Marchinena (27)	2019	293	111 (37.9)	36 (12.3)	35 (11.9)	118 (40.3)	253 (86.3)	91 (31.1)	57 (19.5)	34 (11.6)	35 (11.9)
Lai (28)	2021	89	NA	NA	NA	NA	71 (79.8)	36 (40.4)	18 (20.2)	18 (20.2)	NA

RTP, retrospective; PN, partial nephrectomy; RN, radical nephrectomy; NOS, Newcastle-Ottawa Scale; NA, not applicable; SFI, sinus fat invasion; PFI, perinephric fat invasion; RVI, renal vein invasion; FI, fat invasion; PSI, pelvicaliceal system invasion.

*Median (IQR).

**Mean (range).

### Cancer-Specific Survival of Different Invasion Patterns

**Figure 3:**
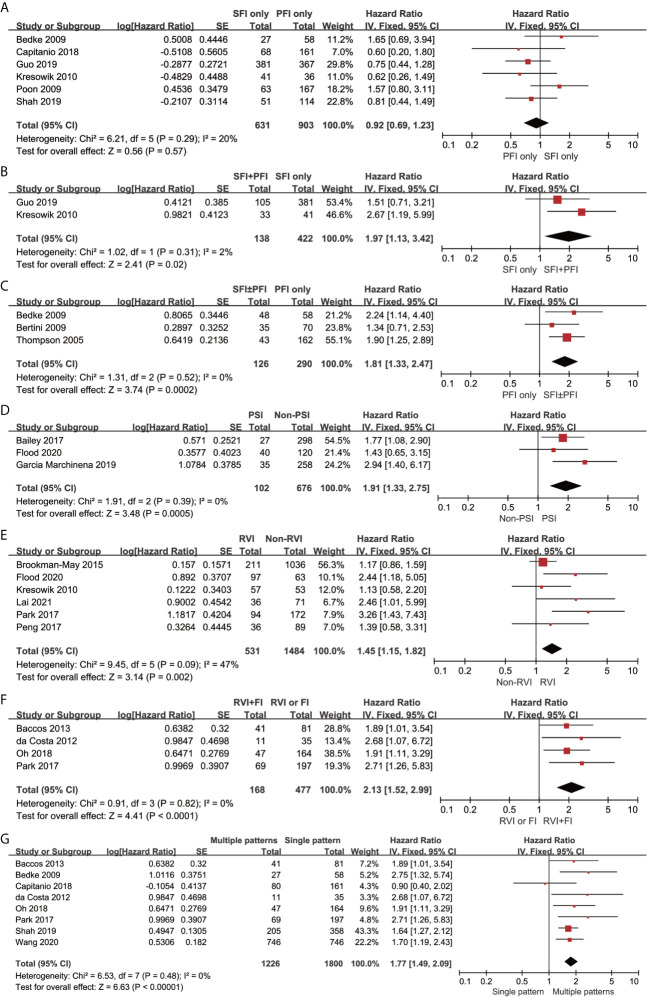
Forrest plots of hazards ratio (HR) evaluating the significant predictors of cancer-specific survival for different pT3a renal tumor invasion patterns. **(A)**: SFI only vs PFI only; **(B)**: SFI + PFI vs SFI only; **(C)**: SFI ± PFI vs PFI only; **(D)**: PSI vs non-PSI; **(E)**: RVI vs non-RVI; **(F)**: RVI + FI vs RVI or FI; **(G)** Multiple patterns vs Single pattern. SFI, sinus fat invasion; PFI< perinephric fat invasion; PSI, pelvicaliceal system invasion; RVI, renal vein invasion; FI, fat invasion.

CSS results were available from 19 studies for different tumor invasion patterns. Although the pooled results revealed that SFI only (n = 631) had comparable CSS to PFI only (n = 903) (HR, 0.92; 95% CI, 0.69–1.23; p = 0.57; I^2^ = 20%; **Figure 3A**), SFI + PFI (n = 138) was associated with inferior CSS as compared to SFI only (n = 422) (HR, 1.97; 95% CI, 1.13–3.42; p = 0.02; I^2^ = 2%; **Figure 3B**). SFI ± PFI (n = 126) showed inferior CSS as compared to PFI only (n = 290) (HR, 1.81; 95% CI, 1.33–2.47; p = 0.0002; I^2^ = 0%; **Figure 3C**). The pooled results revealed that patients with PSI (n = 102) had inferior CSS as compared to those without PSI (n = 676) (HR, 1.91; 95% CI, 1.33–2.75; p = 0.0005; I^2^ = 0%; **Figure 3D**). Patients with RVI (n = 531) had inferior CSS as compared to those without RVI (n = 1484) (HR, 1.45; 95% CI, 1.15–1.82; p = 0.002; I^2^ = 47%; **Figure 3E**), and the coexistence of RVI and fat invasion (FI) (n = 168) showed further deterioration of CSS as compared to RVI or FI (n = 477) (HR, 2.13; 95% CI, 1.52–2.99; p = 0.002; I^2^ = 0%; **Figure 3F**). The multiple invasion pattern (n = 1266) was associated with inferior CSS as compared to single pattern (n = 1226) (HR, 1.77; 95% CI, 1.49–2.09; p < 0.00001; I^2^ = 0%; **Figure 3G**). Using the GRADE approach, the certainty of SFI only vs. PFI only was low, while that of RVI+FI vs. RVI or FI was high. The certainty of SFI+PFI vs. SFI only, SFI ± PFI vs. PFI only, PSI vs. non-PSI, RVI vs. non-RVI and multiple vs. single pattern were all moderate (**Table 3**).

**Table 3:** The overall quality of evidence according to the GRADE (Grading of Recommendations Assessment, Development, and Evaluation) approach.

Comparison	Certainty assessment						No. of patients		Effect		Certainty	Importance
	No. of Studies	Risk of bias	Inconsistency	Indirectness	Imprecision	Large effect	Case	Control	HRs	95% CI		
SFI only vs PFI only	6	not serious	not serious	not serious	serious	No	631	903	0.92	0.69–1.23	⨁⨁⃝⃝ LOW	IMPORTANT
SFI+PFI vs SFI only	2	not serious	not serious	not serious	not serious	No	138	422	1.97	1.13–3.42	⨁⨁⨁⃝MODERATE	IMPORTANT
SFI ± PFI vs PFI only	3	not serious	not serious	not serious	not serious	No	126	290	1.81	1.33–2.47	⨁⨁⨁⃝ MODERATE	IMPORTANT
PSI vs non-PSI	3	not serious	not serious	not serious	not serious	No	102	676	1.91	1.33–2.75	⨁⨁⨁⃝ MODERATE	IMPORTANT
RVI vs non-RVI	6	not serious	not serious	not serious	not serious	No	531	1484	1.45	1.15–1.82	⨁⨁⨁⃝ MODERATE	IMPORTANT
RVI+FI vs RVI or FI	4	not serious	not serious	not serious	not serious	Yes	168	477	2.13	1.52–2.99	⨁⨁⨁⨁ HIGH	IMPORTANT
Multiple vs Single pattern	8	not serious	not serious	not serious	not serious	No	1226	1800	1.77	1.49–2.09	⨁⨁⨁⃝ MODERATE	IMPORTANT
Node N1 vs N0/x	6	not serious	serious	not serious	not serious	No	398	1912	1.71	1.17–2.5	⨁⨁⃝⃝ LOW	IMPORTANT
Metastases M1 vs M0	8	not serious	not serious	not serious	not serious	Yes	726	1844	3.36	2.88–3.91	⨁⨁⨁⨁ HIGH	IMPORTANT
Sarcomatoid Yes vs No	10	not serious	not serious	not serious	not serious	Yes	436	3185	2.09	1.78–2.46	⨁⨁⨁⨁ HIGH	IMPORTANT
Fuhrman grade III or IV vs II or I	8	not serious	not serious	not serious	not serious	Yes	1737	1065	2.7	2.18–3.34	⨁⨁⨁⨁ HIGH	IMPORTANT
Necrosis Yes vs No	6	not serious	not serious	not serious	not serious	No	640	699	1.96	1.54–2.49	⨁⨁⨁⃝ MODERATE	IMPORTANT
Size >7cm vs ≤ 7cm	5	not serious	not serious	not serious	not serious	No	1571	1811	1.77	1.46–2.15	⨁⨁⨁⃝ MODERATE	IMPORTANT
Margin status positive vs negative	6	not serious	not serious	not serious	not serious	Yes	28	1154	7.61	4.12–14.04	⨁⨁⨁⨁ HIGH	IMPORTANT
LVI vs non-LVI	3	not serious	not serious	not serious	not serious	No	159	290	1.11	0.69–1.8	⨁⨁⃝⃝ LOW	IMPORTANT

### Cancer-Specific Survival of Different Pathological Features

**Figure 4:**
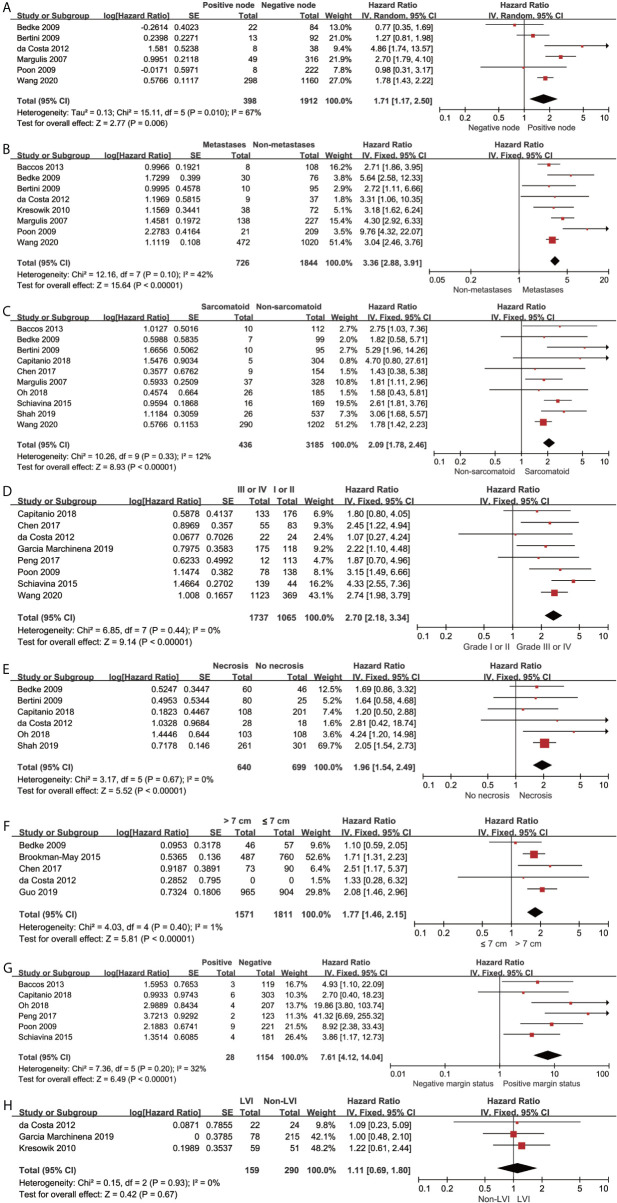
Forrest plots of Hazards ratio (HR) evaluating the significant predictors of cancer-specific survival for different pathological features of pT3a. **(A)**: lymph node involvement; **(B)**: distant metastases; **(C)**: sarcomatoid differentiation; **(D)**: Fuhrman grade (III, IV vs I, II); **(E)**: tumor necrosis; **(F)**: tumor size (>7 cm vs ≤ 7cm); **(G)**: positive margin status; **(H)**: lymphovascular invasion.

The pooled results revealed that lymph node involvement (n = 398; HR, 1.71; 95% CI, 1.17–2.50; p = 0.006; I^2^, 67%; **Figure 4A**), distant metastases (n = 726; HR, 3.36; 95% CI, 2.88–3.91; p < 0.00001; I^2^ = 42%; **Figure 4B**), sarcomatoid differentiation (n = 436; HR, 2.09; 95% CI, 1.78–2.46; p < 0.00001; I^2^ = 12%; **Figure 4C**), Fuhrman grade III or IV (n = 1737; HR, 2.70; 95% CI, 2.18–3.34; p < 0.00001; I^2^ = 0%; **Figure 4D**), necrosis (n = 640; HR, 1.96; 95% CI, 1.54–2.49; p < 0.00001; I^2^ = 0%; **Figure 4E**), tumor size >7 cm (n = 1571; HR, 1.77; 95% CI, 1.46–2.15; p < 0.00001; I^2^ = 1%; **Figure 4F**), and positive margin (n = 28; HR, 7.61; 95% CI, 4.12-14.04; p < 0.00001; I^2^ = 32%; **Figure 4G**) were associated with inferior CSS. The lymphovascular invasion (LVI) (n = 159; HR, 1.11; 95% CI, 0.69-1.80; p = 0.67; I^2^ = 0%; **Figure 4H**) was not a predictor of inferior CSS. Using the GRADE approach, the certainty of lymph node involvement and LVI were low. Tumor size >7 cm and necrosis showed moderate certainty. The certainty of metastases, sarcomatoid differentiation, Fuhrman grade III or IV, and positive margin status were all high.

### Sensitivity Analysis

**Figure 5:**
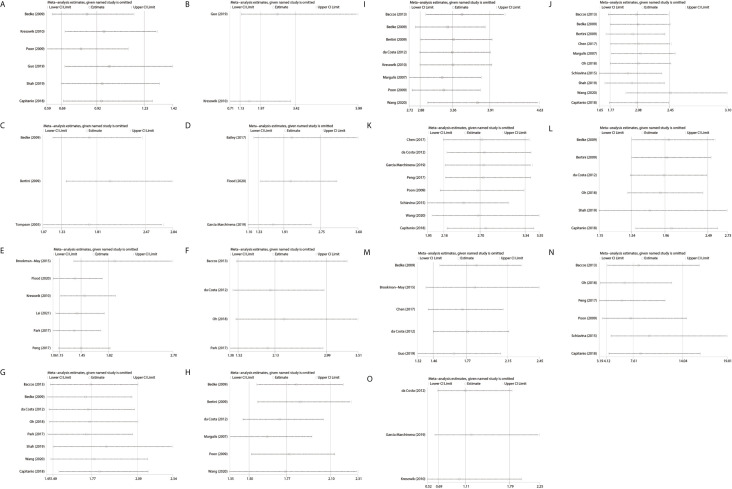
Sensitive analysis of the included studies by one-removed analysis. **(A)**: SFI only vs PFI only; **(B)**: PFI + SFI vs SFI only; **(C)**: SFI ± PFI vs PFI only; **(D)**: PSI vs non-PSI; **(E)**: RVI vs non RVI; **(F)**: RVI + FI vs RVI or FI; **(G)**: multiple vs single pattern; **(H)**: lymph node involvement; **(I)**: distant metastases; **(J)**: sarcomatoid differentiation; **(K)**: Fuhrman grade (III, IV vs I, II); **(L)**, tumor necrosis; **(M)**: tumor size (> 7 cm vs ≤ 7 cm); **(N)**, positive margin status; **(O)**, lymphovascular invasion; SFI, sinus fat invasion; PFI, perinephric fat invasion; PSI, pelvicaliceal system invasion; RVI, renal vein invasion; FI, fat invasion.

The sequential omission of a single study was conducted to test the stability of pooled results. The merged HRs for CSS did not significantly change, which revealed the robustness of the results. (**Figure 5**)

### Publication Bias

The evaluation of publication bias was conducted using Egger’s test only when there were 10 or more included studies. There was no significant publication bias in CSS study of sarcomatoid differentiation (Egger’s test p = 0.232).

## Discussion

Classifying tumors from the surgical perspective and optimizing prognostic discrimination are the cardinal principles in the refinements of the TNM system as T3a renal tumor contains a wide range of four patterns of extrarenal extension regardless of tumor diameter and is confirmed by postoperative pathology in general. Nevertheless, the accuracy and rationality of T3a classification have been questioned in the context of inconsistency of individual oncological outcomes reported in the last fifteen years (3, 16, 18, 21). In the current study, we integrated the available clinical evidence and experience by conducting this systematic review and quantitative synthesis.

The major findings of the current study are the following: First, the moderate-certainty evidence suggests that SFI + PFI was associated with inferior CSS as compared to SFI only. The low-certainty evidence of comparable CSS between SFI only and PFI only and the moderate-certainty evidence of inferior CSS of SFI ± PFI compared to PFI only further support the above findings. Several studies, which merged SFI only and SFI + PFI into a single group given the oncological equipoise derived from their cohorts, may be imprecise (2, 15). Second, moderate-certainty evidence suggests that the presence of PSI indicated significantly poor oncological outcome, with a 1.91 times increased risk of cancer-specific mortality (CSM). Although numerous studies have highlighted the adverse effect of PSI on oncological outcomes, the independent prognostic value of PSI has been excluded from the second to seventh edition of AJCC TNM system (26, 27, 29–31). Palapattu et al. reported a strong relationship between PSI, lymph node invasion and distant metastases (32). Third, high-certainty evidence suggests that the concomitance of RVI and FI significantly increased the risk of deterioration of CSS as compared to RVI or FI supported the finding that multiple invasion patterns translated into moderate-certainty evidence of significantly decreased CSS. However, most contemporary studies that reported the prognostic heterogeneity of T3a RCC failed to comprehensively explore the survival difference among the various combinations. A precise-risk grade of CSS for different invasion patterns, including comprehensive combinations, may be useful for further refinements of the TNM system. Finally, high-certainty evidence indicates that distant metastases, sarcomatoid differentiation, high Fuhrman grade and positive margin were the predictors of inferior CSS. Tumor size >7cm and necrosis also increased the risk of deterioration of CSS, which represents the moderate-certainty evidence. The low-certainty evidence suggests that lymph node involvement might increase the risk of CSM and the lymphovascular invasion was indolent in terms of CSS. The comparable CSS between SFI only and PFI only and the indolent impact of lymphovascular invasion on the survival are inconsistent with the EAU guidelines on RCC, which underlines the prognostic value of several anatomical and histological factors (1). This may require further validation due to the low-certainty evidence.

The inevitable risk of bias caused by the type of surgery that might affect the results of the included studies should be highlighted, even though it had been adjusted in the studies, which included patients undergoing PN or RN. Several studies reported comparable CSS for upstaged pT3a PN patients compared to pT3a RN patients (33, 34). However, the significantly smaller tumor size of the PN cohort compared to the tumor size of the RN cohort indicated that organ confined tumors are susceptible to receive PN. Given the absence of the standardized pathological protocol of capsular invasion in the early years, the classification of renal capsular invasion patterns was an unreliable prognostic variable in some previous studies (2, 35). The recommended routine histopathological examination of perirenal fat was conducted on a discounted basis among patients with peripheral renal tumor since the specimens of renal sinus fat were not systematically collected during PN, especially in the context of the PN enthusiasm (36). The aforementioned factors might have led to the underreported frequency of SFI. Grignon et al. noted that pT1b and pT2 renal tumors probably represented a shrinking proportion when the renal sinus was carefully evaluated (37). In the last two decades, TNM staging classification system for renal tumors was refined three times, which may affect the accuracy and manifolds of pT3a and the heterogeneity of study designs despite minor changes.

Given the increasing PN implementation, the realistic concern is the positive margin, which occurs more frequently in patients with aggressive features, including pT2a, pT3a, and grade III-IV (38, 39). Shah et al. reported that positive margin significantly increased the rate of recurrence, especially among patients with aggressive pathological features, including pT2-T3a, high Fuhrman grade, and clear cell histology (39). In their study, recurrence was observed in almost one third of patients who were up staged to pT3a after PN (40). Bensalah et al. found that positive margin did not cause a decrease CSS; however, the fact that the mean tumor size was 3.5 ± 2 cm, and almost 90% of the positive margin cohort were patients with T1–2 RCC made their conclusions not necessarily applicable to patients with pT3a patients (41). Although several studies mentioned the controversial impact of positive margin on the oncological prognosis among patients with localized RCC, according to the current results that identified more than seven times risk of CSM in patients with pT3a RCC and postoperative positive margin compared to those with negative margin, the weak recommendation of EAU guidelines for intensive follow-up of patients with positive margin may be imprecise (1, 42, 43).

Several studies have highlighted the impact of tumor size on CSS for T3a renal tumor (3, 18, 22, 39, 44–48). In light of the agreement of some studies in which a cutoff of 7 cm was recommended as a prognosis prediction for T3a renal tumor and the applicability of the refinement for the current TNM classification, only the results that considered tumor diameter as a binary variable by using a cutoff of 7cm were merged in the current study. We found that patients with pT3a renal tumor > 7 cm experienced an additional 77% risk of CSM, which was consistent with the findings of Brookman-May et al, reporting that tumor size was identified with the highest prediction accuracy by increasing 71% risk of CSM with a 7 cm cutoff (3). Although the tumor size did not result as a predictor of prognosis in several studies, which cannot be ignored, this is not necessarily contrary to our results and should be further analyzed in the context of the design of the studies and evaluation of patients (14, 19). Whether the impact of tumor size on prognosis can induce T3a and T1/T2 reintegration needs further validation. Chevinsky et al. reported that pT3a had significantly inferior RFS compared to pT1/T2 (45). Chen et al. found that patients with pT3a renal cell carcinoma showed poorer disease-free survival (DSS) as compared to pT1a, pT1b, pT2a, and pT2b. However, Yoo et al. reported a comparable CSS and DSS between pT2 and pT3a ≤ 7cm (49).

The results of the current study may be used to guide the follow-up protocols and select patients suitable for adjuvant therapy after nephrectomy. A compact interval of surveillance may be vital for patients with aggressive factors. Although limited evidence suggested that compact postoperative imaging intervals did not result in the early detection of recurrence, which would benefit survival, the EAU guidelines on RCC recommend a risk-based approach to stratify follow-up for different patients, based on the individual aggressive anatomical, histological and clinical factors (1, 50). The S-TRAC trail exhibited superior disease-free survival (DFS) with sunitinib support. The PROTECT study also reported an improved DFS in the intention to treat pazopanib 800mg population (51). Among the highest-risk subpopulation, the ATLAS study found that axitinib translated into a 36% and 27% reduction in risk of a DFS event per investigator and by independent review committee, respectively (52). However, the recent SORCE trial results, which focused on the DFS and overall survival (OS) in patients with an intermediate or high risk of recurrence, failed to offer positive evidence of sorafenib (53). A recent meta-analysis revealed that adjuvant use of tyrosine kinase inhibitors (TKI) did not translate into improved OS, but showed a benefit in DFS in overall and high-risk populations (54). Due to the lack of sufficient evidence that adjuvant therapy with vascular endothelial growth factor receptor (VEGFR) –TKI offers survival benefits for patients with high-risk RCC, the EAU guidelines on RCC do not recommend the adjuvant therapy after nephrectomy (1). However, heterogeneity among the enrolled patients could be the main cause of the negative results (55). In light of the non-strict inclusion criteria of previous studies, supplemental randomized trials are necessary to determine whether patients with aggressive patterns or characteristics of pT3a renal tumors may benefit from adjuvant treatment.

The present study has some limitations. First, the retrospective nature of the included studies inevitably led the selection bias. Second, the inevitable risk of bias caused by the type of surgery might affect the results. Third, most contemporary studies failed to comprehensively explore the survival difference among the various combinations. Fourth, the determination of the patterns of pT3a renal tumor invasion and pathological features were made by different pathologists, probably based on different criteria. Finally, a small sample of some included studies increased the variability of results.

## Conclusion

The current study identified the heterogenicity of pT3a renal tumors. Multiple invasion patterns could translate into a significantly decreased CSS, and SFI only should not be merged with the SFI + PFI group. The presence of PSI or RVI could significantly increase the risk of cancer-specific mortality. Lymph node involvement, distant metastases, sarcomatoid differentiation, necrosis, high Fuhrman grade, positive margin, and tumor size >7cm are the predictors of inferior CSS. The follow-up protocols and postoperative therapies after nephrectomy should be conducted with individuation according to a risk-based approach for stratification based on these aggressive pathological characteristics. External validation and a precise-risk grade of CSS for different invasion patterns, including comprehensive combinations, may be useful for the further refinements of the TNM system.

## Data Availability Statement

The original contributions presented in the study are included in the article/supplementary material. Further inquiries can be directed to the corresponding authors.

## Author Contributions

PG and YW contributed to the design of the study. YH and DW were responsible for literature search. PG and JZ were responsible for data extraction and analysis. PG and ML were responsible for drafting the manuscript. YJ and YL approved the submitted version. All authors contributed to the article and approved the submitted version.

## Funding

This study was supported by the Natural Science Foundation of Beijing Municipality (award no. 7192053).

## Conflict of Interest

The authors declare that the research was conducted in the absence of any commercial or financial relationships that could be construed as a potential conflict of interest.
